# Improving personalised genetic counselling for ***ABCA4***-associated retinopathy: Updated recurrence risk estimates

**DOI:** 10.1515/medgen-2024-2065

**Published:** 2025-02-12

**Authors:** Stéphanie S. Cornelis, Frans P. M. Cremers

**Affiliations:** Radboud University Medical Center Department of Human Genetics Geert Grooteplein Zuid 10 6525 GA Nijmegen The Netherlands; Radboud University Medical Center Department of Human Genetics Geert Grooteplein Zuid 10 6525 GA Nijmegen The Netherlands

**Keywords:** Recurrence risk, Stargardt disease, *ABCA4*, genotype-phenotype correlation, personalized genetic counseling

## Abstract

Stargardt disease type 1 (STGD1) is caused by biallelic pathogenic variants in *ABCA4*. These variants vary in their effect on the resulting protein and the disease phenotype. Not all variant combinations cause disease, which complicates the determination of the recurrence risk of STGD1. Previously, the recurrence risk of STGD1 was estimated by analyzing variants in patient data and using their population variant frequencies in which white patients are overrepresented. Furthermore, assuming that variant effects are independent of genetic ancestry, estimates were made for each gnomAD population. In this article, the effects of missing heritability, *de novo* variants, reduced penetrance of variants and sex/gender are incorporated and discussed.

## Introduction

When people are diagnosed with an inherited retinal disease, they often wonder what the risk is that their (future) children will also develop the disease. Unfortunately, for autosomal recessive *ABCA4-*associated retinopathy (*ABCA4*-AR), the most frequent inherited macular dystrophy which includes Stargardt disease type 1 (STGD1), this is not very straightforward. For many recessive diseases, the recurrence risk is estimated using the population frequency of all pathogenic variants in the corresponding gene. This helps determine the chance that the other biological parent carries a pathogenic variant. For those diseases, all variants generally are fully deleterious and have the same effect. For *ABCA4*-AR, this is incorrect because variants occur on a spectrum of mild, moderate and severe variants. Many variant combinations, like two mild alleles or a mild and a moderate allele, do not cause disease, while the combination of a severe variant (often a null variant) and another pathogenic variant is expected to cause disease [1]. However, in 2018 it was observed that not all mild variants show complete penetrance (CP). Especially c.5603A>T (p.(Asn1868Ile)) is known to show penetrance of only ~5 % in the general population (N1868I^5 %^) and ~65 % within families when a parent also has *ABCA4-*AR (N1868I^65 %^) [2]. This further complicates recurrence risk calculations for this disease.

In 2022, we estimated the recurrence risk for *ABCA4-*AR for the first time for (future) parents of which one parent has an *ABCA4*-AR diagnosis and the other parent is unaffected and has an unknown genotype [3]. To do so, we incorporated the generally accepted genotype-phenotype model [1,4,5] and took into account that the unaffected parent might still develop *ABCA4*-AR later in life when they have a mild|severe or moderate|moderate genotype. Depending on the genotype of the affected parent, the recurrence risk estimates ranged between 0.7 %–3.7 %. This seems to be in accordance with a Dutch observational study where 1.3 % of families with STGD1 showed pseudo dominant inheritance [6]. For our recurrence risk calculations we classified variants from biallelic persons (BAP) reported in literature up to and including 2020 as mild, moderate, and severe. This allowed to estimate the severity of 504 variants for which the severity was previously unknown. This may help in understanding an individual’s clinical presentation, their prognosis, their recurrence risk and possibly their eligibility for future therapies [7,8]. We then created an allele frequency (AF) dataset based on the gnomAD database and the known and estimated genetic ancestries in the BAP dataset to calculate the recurrence risk for a population that resembles the BAP dataset. The combined AFs of mild^CP^ variants (2.5 %) as well as c.5603A>T (4.9 %) were much higher than combined AFs of moderate variants (0.33 %) and the combined AFs of severe variants (0.45 %). Consequently individuals with *ABCA4*-AR are expected to have a genotype with a mild variant approximately four times more often than not. In this article we describe multiple previously unconsidered factors that could impact the recurrence risk and include these to further improve the recurrence risk estimates [9]. To illustrate the impact of these factors on the resulting estimates, adjustments to the average recurrence risk for individuals with a severe and a mild variant based on changes in expected AFs (Table S1 can be viewed on the website: https://www.gfhev.de/zeitschrift-medizinische-genetik/themenschwerpunkte-details?thema=Focus%20on%20Degenerative%20Retinal%20Disorders) are given for the by BAP represented population, which was 1.83 %. Three significant digits are given in the adjusted risk estimates despite a relatively low level of confidence. Final percentages are indicated with two significant digits. The final underestimates and overestimates for the BAP population as well as for gnomAD populations are in the supplemental tables (Tables S1-S8 can be viewed on the website: https://www.gfhev.de/zeitschrift-medizinische-genetik/themenschwerpunkte-details?thema=Focus%20on%20Degenerative%20Retinal%20Disorders).

## The effect of missing heritability mutations

The previous recurrence risk calculations only took into account identified pathogenic *ABCA4* variants. With the development of improved sequencing techniques that now cover the whole *ABCA4* gene [10], the number of unidentified variants is decreasing. Furthermore, in about one third of individuals with a clinical diagnosis of Stargardt disease a genetic diagnosis could not be made and the cause of disease was found in one of 48 other genes [11]. Due to this, the proportion of unidentified pathogenic *ABCA4* alleles currently is estimated to be ~5 % [6]. These alleles likely include (i) structural variants, which are expected to be identified with upcoming techniques such as long-read sequencing and multiplatform discovery (strategies in which DNA and RNA sequencing data are combined) [12,13], (ii) variants influencing transcription regulation, such as in promotor and (unknown) enhancer regions, and (iii) variants that affect splicing in a retina specific manner, which may currently be missed by splice prediction software [14,15] and may be identified through multiplatform discovery as well. In gnomAD, 15 structural variants are reported that were not taken into account previously. If these variants would be assumed to occur equally in different populations, despite the fact that most of these were identified in the African/African-American population, this would account for almost half of the missing heritability. Assuming that these variants are all severe and that the remaining missing variants occur in ratios of mild, moderate, and severe as estimated previously, this would increase the average recurrence risk of *ABCA4*-AR for a child of a parent carrying a severe and a mild variant from 1.83 % to 2.07 % (Table S1.1 can be viewed on the website: https://www.gfhev.de/zeitschrift-medizinische-genetik/themenschwerpunkte-details?thema=Focus%20on%20Degenerative%20Retinal%20Disorders).

## The effect of *de* *novo* mutations

Another effect that was not taken into consideration previously, is the occurrence of *de novo* variants in future generations. In 2012, a *de novo* mutation rate of 1.20 × 10^-8^ per nucleotide per generation was reported by Kong et al. [16]. If we assume that this rate is applicable to *ABCA4*, about 0.008 % of the unaffected parents will pass on a *de novo* mutation in a coding region or at a canonical splice site of *ABCA4*. If all of those variants were pathogenic in a similar distribution as estimated for the other variants in *ABCA4*, the original recurrence risk for a parent carrying a severe and a mild variant would increase less than 0.01 % (Table S1.2 can be viewed on the website: https://www.gfhev.de/zeitschrift-medizinische-genetik/themenschwerpunkte-details?thema=Focus%20on%20Degenerative%20Retinal%20Disorders). Alternatively, we could estimate the increased recurrence risk based on an observational study from Li et al., 2023. This study reports that 3.5 % (with a 95 % confidence interval (CI) of 0.43 %–12 %) of individuals with STGD1 carry *de novo* mutations and shows *ABCA4* to be in the top ten of genes with *de novo* mutations causing inherited eye disease [17]. Therefore, 96.5 % (95 % CI of 88 %–99.57 %) of STGD1 individuals inherited a pathogenic variant that is not *de novo*, which corresponds with the patient group that our recurrence risk calculations were based on, while the remaining STGD1 individuals develop STGD1 due to a *de novo* variant in *ABCA4*. Incorporating this increases the recurrence risk from 2.07 % to 2.14 % (95 % CI of 2.06 %–2.30 %) (Table S1.3 can be viewed on the website: https://www.gfhev.de/zeitschrift-medizinische-genetik/themenschwerpunkte-details?thema=Focus%20on%20Degenerative%20Retinal%20Disorders). The discrepancy between calculations could be in part due to the possibility that *ABCA4* might be more prone to the introduction of *de novo* mutations than average since the introduction of mutations in the genome is not equally distributed. The gnomAD database shows a slightly higher number of observed versus expected variants in *ABCA4* and a small mutation hotspot has been identified covering part of exon 4 and intron 4 of *ABCA4* (GC content: 52 %) [18,19]. Although this region only contains seven known (potentially) pathogenic variants and none of the *de novo* variants identified by Li et al. were located in this region [17], it might still indicate that *ABCA4* is in general susceptible to the introduction of mutations. Furthermore, mutation hotspots are found to be enriched in gene pathways related to sensory perception further indicating that *de novo* variants may be more frequent in *ABCA4* than average in the genome [19]. In the following adjustments, the former *de novo* rate based on Kong et al., was incorporated. At the final recurrence risk estimation at the end of this article, an alternative estimate based on the Li et al. *de novo* rate is described.

## The effect of reduced penetrance

Our previous calculations included the estimated reduced penetrance of the frequent variant c.5603A>T but did not incorporate the reduced penetrance of other mild variants. If we were to include the estimated reduced penetrance of the variants c.3113C>T, c.4253+43G>A, c.5882G>A and c.6089G>A as in Table 1, the adjusted recurrence risk for a parent carrying a severe and a mild variant would decrease from 2.07 % to 1.76 % (Table S1.4 can be viewed on the website: https://www.gfhev.de/zeitschrift-medizinische-genetik/themenschwerpunkte-details?thema=Focus%20on%20Degenerative%20Retinal%20Disorders). Reduced penetrance for the variants c.2588G>C and c.5714+5G>A was not included in this adjustment considering that c.2588G>C is likely not pathogenic and c.5714+5G>A is now expected to show full penetrance [20].

**Table 1:** **Estimated penetrance of mild variants according to Runhart et al., 2019 and Runhart et al. 2020 [21,22].** The 95 % confidence intervals (CI) are based on the observed number of individuals in the Radboudumc for c.4253+43G>A and the LOVD of *ABCA4* for the other variants as well as the expected number of individuals based on the non-Finnish European population in gnomAD as reported in these studies were added.

Variant	Penetrance	95 % CI
c.3113C>T	17 %	[12 %; 22 %]
c.4253+43G>A	39 %	[16 %; 62 %]
c.5882G>A	50 %	[45 %; 55 %]
c.6089G>A	36 %	[24 %; 48 %]

## Corrected recurrence risks including 95 % confidence intervals

Incorporating the aforementioned corrections into the model changes the estimated average recurrence risk for people with a severe and a mild *ABCA4* variant from the originally estimated 1.83 % to 1.76 % (Table S1.4 can be viewed on the website: https://www.gfhev.de/zeitschrift-medizinische-genetik/themenschwerpunkte-details?thema=Focus%20on%20Degenerative%20Retinal%20Disorders). Moreover, when we also incorporate the 95 % confidence intervals of both the reduced penetrance calculations and the gnomAD adjusted AF to the underestimate and the overestimate as described in our original calculations, the recurrence risk for individuals with a severe and a mild *ABCA4* variant is estimated to be between 1.5–2.1 % (Table 2; Tables S1.5-S1.6 can be viewed on the website: https://www.gfhev.de/zeitschrift-medizinische-genetik/themenschwerpunkte-details?thema=Focus%20on%20Degenerative%20Retinal%20Disorders).

**Table 2:** **Adjusted average recurrence risks per parental genotype for the population represented by the BAP dataset.** The total and severe phenotype recurrence risks are based on the sum of AF of mild, moderate, and severe variants. ↓ represents the underestimate including the lower bound of 95 % confidence intervals, adjustments, excluding the additional Variants of Uncertain Significance (VUS) and Likely Pathogenic (LP) variant frequencies. ↑ represents the overestimate including adjustments, the upper bound of 95 % confidence intervals and the additional VUS and LP variant frequencies.

Parental genotype	Total recurrence risk (↓-↑)	Severe phenotype recurrence risk (↓-↑)
Severe|Severe	3.0 % (2.5 %–3.5 %)	0.86 % (0.72 %–1.0 %)
Severe|Moderate	1.9 % (1.6 %–2.3 %)	0.70 % (0.60 %–0.80 %)
Moderate| Moderate	0.86 % (0.72 %–1.0 %)	0.53 % (0.48 %–0.58 %)
Severe|Mild^CP^	1.8 % (1.5 %–2.1 %)	0.43 % (0.36 %–0.51 %)
Severe|N1868I^65 %^	1.7 % (1.4 %–2.0 %)	0.43 % (0.36 %–0.51 %)
Severe|WT	1.5 % (1.2 %–1.8 %)	0.43 % (0.36 %–0.51 %)
Moderate|WT	0.43 % (0.36 %–0.51 %)	0.27 % (0.24 %–0.29 %)
Mild^CP^|WT	0.27 % (0.24 %–0.29 %)	–
N1868I^65 %^|WT	0.17 % (0.16 %–0.19 %)	–
N1868I^5 %^|WT	0.013 % (0.012 %–0.015 %)	–

## Recurrence risk in various populations

In our previous study, the recurrence risks were also calculated per gnomAD population to estimate the recurrence risk per population based on the genetic ancestry of the unaffected parent. Adding the adjustments and the 95 % confidence intervals for gnomAD AFs to the recurrence risks per gnomAD population generally leads to a wider interval between underestimate and overestimate than for the population represented by the BAP dataset (Table 3; Tables S2-S8 can be viewed on the website: https://www.gfhev.de/zeitschrift-medizinische-genetik/themenschwerpunkte-details?thema=Focus%20on%20Degenerative%20Retinal%20Disorders). Of note, since the BAP dataset mainly included individuals who were likely from non-Finnish European ancestry and the adjusted gnomAD AFs were based on estimated genetic ancestry of affected individuals, it is possible that frequent variants from populations other than the non-Finnish European population have erroneously been estimated to be pathogenic, leading to an overestimate. This is especially likely in the African/African-American population where two variants (c.1927G>A & c.2546T>C) categorised as benign/mild have a combined AF of 0.030 and two variants (c.3076T>C and c.4556C>T) with a combined AF of 0.0013 were categorised as moderate/severe. Similarly in the Ashkenazi Jewish population four variants (c.3758C>T, c.4139C>T, c.4594G>A, and c.5056G>A) with several severity categories, had a combined AF of 0.019. This leads to a high recurrence risk in these populations, which is especially high for offspring phenotypes including mild variants. Therefore, it is important to investigate if the predicted severity of the most frequent variants per severity group per population are categorised correctly, since these variants have the highest impact on the recurrence risk calculations. Furthermore, since most known mild variants were identified in countries where people with a non-Finnish European ancestry are overrepresented, it will be of interest to investigate if there are currently unknown mild variants in all the remaining gnomAD populations that might show reduced penetrance, such as the aforementioned benign/mild variants in the African/African-American population. Conversely, pathogenic variants that were absent or underrepresented in the BAP population and that are frequent in non-white populations are likely excluded in the estimates and could falsely indicate a low recurrence risk in some populations.

**Table 3:** **Adjusted average recurrence risks for parents with a severe|mild^CP^ genotype per gnomAD population.** ↓ represents the underestimate including adjustments and the lower bound of 95 % confidence intervals. ↑ represents the overestimate including adjustments and the upper bound of 95 % confidence intervals.

gnomAD population	Total recurrence risk (↓-↑)	Recurrence risk for severe phenotype (↓-↑)
African/African-American	4.7 % (3.7 %–5.7 %)	0.52 % (0.33 %–0.76 %)
Latino/Admixed American	1.6 % (1.2 %–1.7 %)	0.53 % (0.38 %–0.66 %)
Ashkenazi Jewish	2.7 % (2.0 %–3.3 %)	0.39 % (0.26 %–0.52 %)
East Asian	1.5 % (1.1 %–1.5 %)	0.34 % (0.19 %–0.46 %)
European (Finnish)	1.3 % (0.65 %–2.0 %)	0.15 % (0.10 %–0.19 %)
European (non-Finnish)	1.6 % (1.3 %–2.0 %)	0.44 % (0.38 %–0.52 %)
South Asian	1.3 % (1.0 %–1.4 %)	0.28 % (0.19 %–0.37 %)

## Sex/gender effect on recurrence risk

In 2024, an overrepresentation of women among the group of individuals with a mild and a severe variant was identified with a general ratio of 0.59 women to 0.41 men [20]. This means that among individuals with a severe and a mild variant, women are 1.44 times more likely to be affected by STGD1 than men. It is unknown if this effect is caused by a difference in sex, which usually refers to a person’s biological characteristics, or a difference in gender, which usually refers to a person’s identity and the sociocultural expectations of behaviour associated with a given sex. Therefore, further comments on sex or gender only applies to people for which gender correlates with the sex assigned at their time of birth and only include male or female. The overrepresentation effect of women could be incorporated into the recurrence risk for offspring of which the sex is known by multiplying the risk of having offspring with a mild and a severe variant by 0.59/0.5 for women and by 0.41/0.5 for men (sheets 4–6 in all supplemental tables). We expect that the recurrence risk for intersex and transgender individuals will likely lie between these ranges.

## Final recurrence risk estimation

Based on the adjustments described above, including the genomic *de novo* mutation rate [16], the recurrence risk likely lies within the range of 1.2 %–2.6 % for individuals in a population similar to the population represented by the BAP dataset, which from all our calculations best resembles populations in North America and Europe. Importantly, women are at an increased risk compared to men. Incorporating the *ABCA4*-specific mutation rate from Li et al., 2023, widens this range to 1.2 %–2.9 %, with an average risk of 1.9 %.[17].

## Further limitations

In this article we considered factors that could impact the recurrence risk estimates of *ABCA4-*AR. However, at least five more factors could not be incorporated in the adjusted model: (i) Due to population stratification variant homozygosity is likely more frequent than assumed based on the BAP variant frequencies, considering that in some geographic regions certain variant frequencies will be higher leading to a higher homozygosity occurrence. This could have overestimated the number of moderate variants, leading to an overestimate of the recurrence risk; (ii) Variant frequencies were assumed to be non-complex, apart from a few known recurrent complex variants. However, these are particularly well known among populations in Europe and the USA, meaning that especially for gnomAD populations other than the ones with non-Finnish European ancestry, the calculations likely overestimate the recurrence risk. Currently gnomAD has an online tool available to check co-occurrence of exonic variants [23]. Checking the co-occurrence of frequent variants can further improve the estimates; (iii) Reduced penetrance of mild variants in *ABCA4-*AR has only been estimated based on prevalence, while cumulative incidence would likely have been more accurate. This could have led to an underestimate of the recurrence risk especially for individuals with mild variants that are known to cause late-onset STGD1; (iv) *ABCA4* variant severity likely describes a spectrum. In the used genotype-phenotype model this spectrum has been reduced to the three categories mild, moderate and severe. Therefore, known mild variants might not all have the exact same effects, impacting the analyses in which these were used as one category. Consequently, not all variants categorized here as mild, moderate or severe will cause *ABCA4-*AR in the expected combinations of mild|severe and moderate|moderate. In addition, some mild|moderate combinations may cause *ABCA4*-AR; (v) Finally, in the analysis of this article we only included *ABCA4* variants that were published before 2021. More *ABCA4* variants have been identified by now and will continue to be identified in the future. Incorporating those variants and their updated severity classifications will improve the accuracy of the recurrence risk calculations, especially in populations which are underrepresented in literature before 2021.

## Conclusion

Considering that most of these last assumptions have likely led to an overestimation of the recurrence risk in these analyses, it is likely that that the recurrence risk for *ABCA4-*AR is well below 3 % for most individuals. Whether the recurrence risk is much higher for individuals of which the unaffected parent is of African/African American descent (up to 6.5 % for women) should be investigated further to clarify if *ABCA4*-AR is more frequent in individuals of African descent or whether the pathogenicity of (some) frequent variants in people of African descent have been overestimated. Moreover, we currently lack understanding of the nature and thus prevalence of genetic and/or non-genetic modifiers for *ABCA4*-AR, which could be very different between populations. In conclusion, incorporating additional factors that impact the recurrence risk of *ABCA4-*AR has not led to a major change in the estimated recurrence risk of the disease. The most important next step to improve *ABCA4-*AR recurrence risk estimates is to identify the pathogenicity and severity of individual *ABCA4* variants and especially in populations and subpopulations that have been underrepresented in literature so far.

## Supplemental Material

The average recurrence risk estimates with all adjustments are in sheet five of each file. Gender/sex specific risks are included in columns H and I of sheets 5, 6 and 7 of each file.

**Table S1 Recurrence risk estimates based on the BAP population**

**Table S2 Recurrence risk estimates based on the gnomAD African/African American population**

**Table S3 Recurrence risk estimates based on the gnomAD Latino/Admixed American population**

**Table S4 Recurrence risk estimates based on the gnomAD Ashkenazi Jewish population**

**Table S5 Recurrence risk estimates based on the gnomAD East Asian population**

**Table S6 Recurrence risk estimates based on the gnomAD Finnish population**

**Table S7 Recurrence risk estimates based on the gnomAD non-Finnish European population**

**Table S8 Recurrence risk estimates based on the gnomAD South Asian population**

**The tables S1-S8 can be viewed on the website:** https://www.gfhev.de/zeitschrift-medizinische-genetik/themenschwerpunkte-details?thema=Focus%20on%20Degenerative%20Retinal%20Disorders

## Supplementary Material

Supplementary Material
